# Architecture of Inherited Susceptibility to Colorectal Cancer: A Voyage of Discovery

**DOI:** 10.3390/genes5020270

**Published:** 2014-03-27

**Authors:** Nicola Whiffin, Richard S. Houlston

**Affiliations:** Molecular and Population Genetics Team, Genetics and Epidemiology, The Institute of Cancer Research, Sutton, SM2 5NG, UK; E-Mail: richard.houlston@icr.ac.uk

**Keywords:** colorectal cancer, genetics, susceptibility

## Abstract

This review looks back at five decades of research into genetic susceptibility to colorectal cancer (CRC) and the insights these studies have provided. Initial evidence of a genetic basis of CRC stems from epidemiological studies in the 1950s and is further provided by the existence of multiple dominant predisposition syndromes. Genetic linkage and positional cloning studies identified the first high-penetrance genes for CRC in the 1980s and 1990s. More recent genome-wide association studies have identified common low-penetrance susceptibility loci and provide support for a polygenic model of disease susceptibility. These observations suggest a high proportion of CRC may arise in a group of susceptible individuals as a consequence of the combined effects of common low-penetrance risk alleles and rare variants conferring moderate CRC risks. Despite these advances, however, currently identified loci explain only a small fraction of the estimated heritability to CRC. It is hoped that a new generation of sequencing projects will help explain this missing heritability.

## 1. Introduction

Colorectal cancer (CRC) is the third most common cancer worldwide with half a million new individuals diagnosed annually [1]. In the UK CRC affects ~40,000 individuals and is responsible for ~16,000 deaths each year (Cancer Research UK, 2013) amounting to a life-time risk of 5%–6%. It is now an established fact that inherited susceptibility has an important role in predisposition to CRC. The earliest evidence for this came from epidemiological studies in the 1950s which showed a two- to three-fold increased risk of CRC in first degree relatives of patients [2]. Subsequent studies have identified a number of CRC susceptibility genes. These discoveries have greatly increased our understanding of the mechanisms underlying CRC biology and have provided promising targets for therapeutic intervention. Moreover, the ability to identify individuals at increased risk of CRC is of important clinical relevance.

## 2. Early Models of Genetic Susceptibility

Large families with multiple individuals affected by CRC have long been reported in the clinic. It was not until the late 1950s, however, that epidemiological studies attempted to quantify this familial clustering by comparing the incidence of CRC in first degree relatives (FDRs) of cases to control groups [3,4,5]. Recent analysis estimates an approximate two-fold increase in risk in FDRs [2]. This risk increases further to four-fold when the relative is diagnosed with early-onset CRC (<45 years of age), indicative of colorectal tumours developing in genetically susceptibly individuals at an earlier age.

In 1969 Ashley [6] proposed that colonic cancer development could be ascribed to a series of carcinogenic “hits” on normal intestinal mucosa cells. He further noted that the number of necessary “hits” was lower for genetically susceptible individuals with the Mendelian predisposition syndrome familial adenomatous polyposis (FAP). In the same year, DeMars [7] suggested that FAP, along with other apparently autosomal dominant syndromes, is autosomally recessive at the cellular level; individuals with a germline mutation in one allele of a tumour suppressor gene develop cancer as a result of subsequent somatic mutations in the other gene copy.

Anderson [8], in 1974, made the first argument for a polygenic mechanism to cancer susceptibility based on the increased risk in FDRs of cancer patients being limited to two- to three-fold. He stated these results were “not indicative of strong genetic effects” and rather suggested a mechanism involving many genes with small effects acting in concert with environmental factors with larger effects. Whilst there is growing evidence to suggest that his conclusion is, at least in part, correct, the reasoning behind this statement is flawed as the relative risks associated with FDRs are likely to be underestimated. This is because calculations of relative risks typically include both sporadic and genetically susceptible cases that are then compared to the general population which, to compound the problem, also contains individuals that are genetically susceptible to CRC.

## 3. Identification of Rare High-Penetrance Susceptibility Alleles

Evidence for Mendelian transmission of CRC was first provided by reports of large families with CRC segregating in a dominant fashion. Perhaps the most notable case report is “family G” first described in 1913 by Warthin and subsequently revisited by Lynch and Krush in 1971 [9]. This family of over 650 blood relatives provided scientists with one of the longest, most detailed cancer genealogies in the world and was instrumental in establishing the syndrome of hereditary non-polyposis colorectal cancer (HNPCC or Lynch syndrome).

Family based genetic linkage and positional cloning studies in the late 1980s and early 1990s led to the identification of numerous CRC susceptibility genes (Table 1). The *APC* gene on chromosome 5 was the first gene to be shown to be associated with CRC susceptibility when it was identified as mutated in FAP patients [10,11,12,13,14]. Subsequently, mutations in genes of the mismatch repair (MMR) pathway, particularly *MSH2*, *MLH1* and MSH6, the TGF-β signalling pathway genes, *SMAD4* and *BMPR1A*, and the serine/threonine kinase gene STK11, were revealed as the causes of HNPCC [15,16,17,18,19,20,21,22,23,24,25,26], Juvenile Polyposis syndrome (JPS) [27,28] and Peutz-Jeghers syndrome (PJS) [29] respectively. Risk alleles in these genes are rare (<0.1%) and confer a >10 fold increase in risk of CRC. These genes are tumour suppressors conforming to DeMars’ “two-hit” model of cancer susceptibility through an apparently dominant mode of inheritance. The clinical utility of testing for high penetrance mutations in these genes has long been established and identification of individuals with such mutations has been shown reduce CRC incidence through prevention strategies and screening [30,31].

**Table 1:** Colorectal cancer predisposition syndromes and associated high-penetrance mutations.

Gene(s)	Syndrome	Risk in mutation carriers	Mode of inheritance	References
APC	FAP	90% by age 45	Dominant	[10,11,12,13,14]
Mismatch repair (MLH1/MSH2/MSH6/PMS2)	HNPCC/Lynch syndrome	40%–80% by age 75	Dominant	[15,16,17,18,19,20,21,22,23,24,25,26,32,33]
SMAD4/BMPR1A	JPS	17%–68% by age 60	Dominant	[26,27]
STK11	PJS	39% by age 70	Dominant	[28]
MUTYH	MYH-associated polyposis	35%–53%	Recessive	[34,35]
POLD1/POLE	Oligopolyposis		Dominant	[36]

## 4. More Recent Models of Genetic Susceptibility

Studies examining the difference in CRC development between monozygotic and dizygotic twins estimated that ~35% of CRC could be ascribed to a genetic predisposition [37]. However, <10% of all CRC can be accounted for by germline mutations in *APC* and the MMR genes and crucially ~70% of the familial risk of CRC remains unexplained [38].

**Figure 1:**
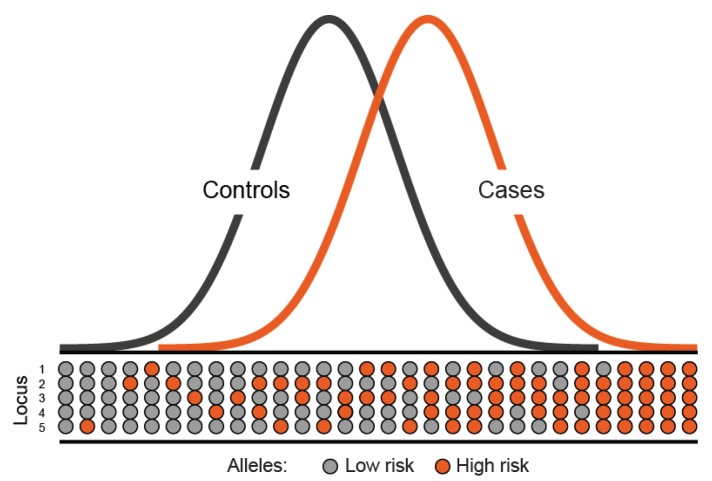
Polygenic model of disease susceptibility. The distribution of risk alleles in both cases and controls follows a normal distribution. However, cases have a shift towards a higher number of high risk alleles.

Over the past 20 years, extensive efforts to identify additional, highly penetrant cancer susceptibility genes for CRC through conventional linkage scans have met with limited success [39,40]. This strongly implies that any additional high penetrance CRC gene will individually account for only a small proportion of the familial risk. Statistical modelling of the pattern of familial occurrence of CRC after exclusion of known high-risk genes suggests that much of the inherited susceptibility is likely to be polygenic with the co-inheritance of multiple genetic variants, each with a modest individual effect, causing a wide range of risk in the population (Figure 1).

Over the past two decades candidate gene studies have identified rare moderately-penetrant risk alleles (minor allele frequency (MAF) < 2%; relative risks (RRs) > 2.0) and more recent genome-wide association studies (GWAS) have identified common, low-penetrance alleles (MAF > 10%; OR < 1.5). In reality, these variants are likely to occur as a continuum and the separate classes of risk alleles merely reflect the subgroups detectable using current methodologies.

## 5. Rare, Moderately-Penetrant Disease-Causing Variants

The “rare-variant” hypothesis suggests that much of the remaining heritability could be due to the combined effect of rare, moderately-penetrant risk alleles [41]. These variants are hypothesised to act independently and to confer modest, but detectable, increases in risk.

Attempts to identify this class of disease allele have mainly been through resequencing candidate genes in affected families, the success of which has been hampered by our limited knowledge of tumour biology. The identification of the missense variant, APC I1307K, carried by ~6% of Ashkenazi Jews and conferring around a two-fold increase in risk of CRC [42] and the more recent discovery of the functional promoter variant -93G>A of MLH1, shown to predispose to microsatellite unstable CRC [43], represent rare successes of this approach.

*A priori* rare disease-causing alleles are likely to act in a dominant fashion; however, functional variants in the base-excision repair gene *MUTYH* provide an example of a recessive model of inheritance [34]. Biallelic or compound heterozygosity of the G396D and Y179C mutations in *MUTYH*, which are carried by around 1%–2% of the UK population, confers a CRC risk comparable to that seen in carriers of germline MMR mutations [35].

Mechanistically these variants are likely to be directly causal. For the variants in *MUTYH*, insights into biological basis of susceptibility came from the method in which they were discovered; a FAP family with no apparent *APC* mutation was found to possess a mutator phenotype reflective of defective base excision, resulting in somatic mutation of *APC* and other genes [34,44]. In contrast, the *APC* I1307K T>A variant appears to increase replication errors in *APC* through generation of a run of eight adenines [42].

## 6. Identification of Common Low-Penetrance Alleles

The “common-variant, common-disease” hypothesis states that a substantial proportion of the remaining risk is likely to be accounted for by the summation of numerous low-penetrant genetic variants, each with a relatively high frequency in the population [45]. These variants have more subtle effects on gene regulation and predominantly reside within non-coding regions of the genome. Each individual variant is associated with only a modest increase in risk; however, collectively they may confer a substantial increase in disease susceptibility. These alleles rarely cause multiple cases in families and therefore cannot be detected through genetic linkage studies [46]. Initial attempts to identify this class of allele through candidate gene association studies were based on small case-control series and had little success; any proposed variants were not successfully validated in subsequent studies.

Genome-wide association studies (GWAS), typically based on genotyping of 300,000 to over 1 million SNPs, have proved to be a powerful approach in identifying common, low penetrance susceptibility loci for CRC without prior knowledge of location and function.

**Figure 2:**
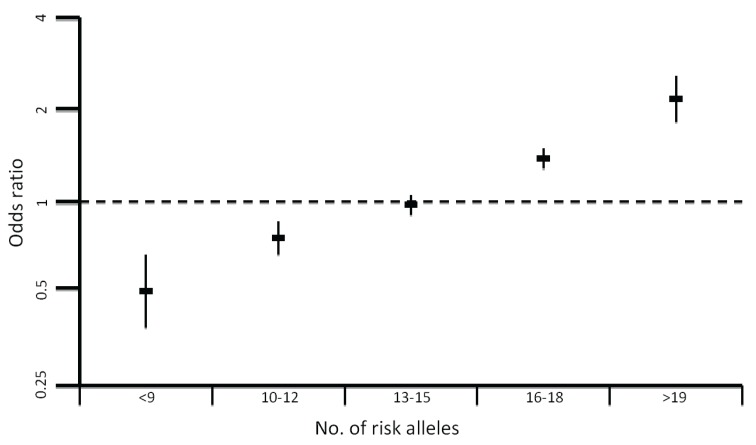
Plot showing the increase in odds ratio for colorectal cancer with an increasing number of risk alleles.

Since the first CRC GWAS in 2007, 18 CRC susceptibility loci have been identified in European populations (Table 2) [47,48,49,50,51,52,53]. While each individual risk allele confers only a small relative risk (1.06 < OR < 1.26), the SNPs are common (MAF > 10%) and hence contribute significantly to the overall incidence of CRC. Moreover, by acting in concert they can impact significantly on an individual’s risk of developing CRC (Figure 2) [54]. The design of association studies is advantageous as large numbers of unrelated case and control samples may be readily obtained, providing adequate power to detect loci with relatively small effects. This is in contrast to the difficulty in recruiting the extensive pedigrees required for linkage studies.

Importantly, few genes implicated in GWAS were previously evaluated in candidate gene studies, highlighting the importance of such an agnostic approach for gene discovery and understanding of CRC aetiology. None of the currently identified loci, for example, are involved in DNA repair, which is the principle pathway underscoring high-penetrance CRC susceptibility. Interestingly, five of the loci discovered to date are within or near to genes involved in the TGF-β signalling pathway [47,50]. This pathway has already been implicated in pathogenesis of CRC, as the dominant CRC predisposition syndrome JPS is caused by high penetrance mutations in the TGF-β family genes *SMAD4* and *BMPR1A* [13,14].

**Table 2:** Loci identified as associated with colorectal cancer through genome-wide association studies and meta-analyses.

Locus	Nearest Gene(s)	GWAS tagSNP	Location	Risk Allele	Alt Allele	RAF
1q41	*DUSP10*	rs6691170	222,045,446	T	G	0.40
3q26.2	*TERC*, *MYNN*	rs10936599	169,492,101	C	T	0.75
6p21.2	*CDKN1A*	rs1321311	36,622,900	T	G	0.21
8q23.3	*EIF3H*	rs16892766	117,630,683	C	A	0.09
8q24.21	*MYC*	rs6983267	128,413,305	G	T	0.52
10p14	*GATA3*	rs10795668	8,701,219	G	A	0.67
11q13.4	*POLD3*	rs3824999	74,345,550	C	A	0.47
11q23.1	*FLJ45803*	rs3802842	111,171,709	C	A	0.27
12q13	*DIP2B*, *ATF1*	rs11169552	51,155,663	C	T	0.75
14q22.2	*BMP4*	rs4444235	54,410,919	C	T	0.48
15q13.3	*SCG5, GREM1*	rs4779584	32,994,756	T	C	0.19
16q22.1	*CDH1*	rs9929218	68,820,946	G	A	0.71
18q21.2	*SMAD7*	rs4939827	46,453,463	T	C	0.53
19q13.11	*RHPN2*, *GPATCH1*	rs10411210	33,532,300	C	T	0.90
20p12.3	*BMP2*	rs961253	6,404,281	A	C	0.37
		rs4813802	6,699,595	G	T	0.34
20q13.33	*LAMA5*	rs4925386	60,921,044	C	T	0.68
Xp22.2	SHROOM2	rs5934683	9,751,474	T	C	0.56

## 7. Functional Effects of GWAS Loci

Elucidating the basis of association at common low-penetrance loci represents a significant challenge. The tagging SNPs (tagSNPs) used in GWAS are not necessarily strong candidates for being causal and were instead chosen to capture variation across large genomic regions. Hence, establishing which of a set of highly correlated variants in linkage disequilibrium (LD) with the tagSNP is the true causal variant is a challenge. In addition, many GWAS loci map to non-coding regions or gene deserts, suggesting the true cause of the association at these regions is through subtle effects on gene expression rather than changes to protein coding sequence.

Fine mapping of CRC loci is still in its infancy with most studies attempting only to narrow down the location of a likely functional variant through imputation or re-sequencing [55,56,57]. These studies suggest candidate variants but very few functional studies have been carried out to test these assertions. To date, in only four regions has a SNP been proposed as the likely functional candidate and a mechanism of action suggested (8q23.3 [58], 8q24.21 [59,60], 11q23.1 [61], 18q21.1 [62]). The most intriguing of these regions is 8q24.21, which has pleiotropic effects on cancer susceptibility, also harbouring risk loci for breast [57], ovarian [63], bladder [64], CLL [65] and multiple independent loci for prostate cancer [66,67,68,69]. This is in contrast to most associations found to date which appear to be disease specific. The rs6983267 risk SNP is associated with both prostate and colorectal cancers and lies within an evolutionarily conserved region. The two alleles of rs6983267 show differential binding of the TCF4 transcription factor [59] to an enhancer element that has been shown to physically interact with the *MYC* gene promoter [60]. *MYC* is amplified or over-expressed in multiple cancer types leading to up-regulation of many genes controlling cell proliferation, so it is predicted that variation at this locus acts through subtle effects on *MYC* gene expression. The risk allele of rs6983267 has also been suggested as a marker of worse prognosis in CRC patients [70]. The strongest CRC GWAS association is at 18q21.1 (RR = 1.26) within the *SMAD7* gene, which acts as an antagonist of the TGF-β signalling pathway. Resequencing of the 17 kb region of linkage disequilibrium surrounding the GWAS tagSNP rs4939827 identified a novel variant, termed Novel1 (rs58920878), which was shown to affect *SMAD7* expression [62].

## 8. Impact of Common Variation on CRC Risk

Collectively, the currently identified risk loci explain only ~6% of the overall familial risk of CRC. This estimate is likely conservative as the effect of the causal variant at each locus is expected to be greater than the association detected through a GWAS tagSNP. In addition, as evinced by the 14q22 (*BMP4*) [52] association multiple risk variants may exist at each locus, including low-frequency variants with significantly larger effects. Moreover, epistatic interactions may exist between common risk loci, which could make the contribution of low-penetrance susceptibility alleles much higher. Such interactions remain difficult to detect due to substantial multiple testing penalties, and existing studies suggest the effects of most common low-penetrance alleles seem to be independent. In addition, interactions of these alleles with epigenetic regulation or environmental factors may lead to a greater increase in disease risk. For example the *MLH1*-93G>A polymorphism has been shown to be associated with increased methylation of the *MLH1* promoter [71]. Another important consideration is the possible modification of the effect of a low-penetrance allele by the presence of a high-penetrance mutation. The only evidence to support this assertion in CRC is a small study implicating the 8q23.3 and 11q23.1 CRC SNPs as modifiers of CRC risk in MMR mutation carriers [72]. Although there was initial hope for the use of low penetrance variants in the clinic, the small proportion of familial risk explained and the apparent lack of epistatic interactions between the variants leads to them being of low predictive power. The increased risk associated with having a high number of risk alleles (Figure 2) has the prospect of identifying individuals in the general population who might benefit from earlier screening [54].

## 9. Identifying Novel CRC Susceptibility Loci

It is unlikely that there are any common CRC risk loci with appreciable MAF (>10%) and with relative risks >1.1 that remain to be uncovered given the size of existing GWAS studies. Small variant effect sizes combined with stringent thresholds for establishing statistical significance and financial constraints on the number of variants which can be followed up constrain GWAS study power. However, many GWAS have long tails of association with alleles of increasingly small effect, suggesting much of the remaining susceptibility may be embodied in a multitude of common risk alleles. Larger GWAS studies combining multiple phases and tens of thousands of cases may identify many more of these susceptibility loci, although the effect of these on CRC risk is likely to be minimal. Such studies have been conducted in both breast [73] and prostate [74] cancer identifying 41 and 23 novel risk loci respectively.

Commercial arrays used for GWAS capture a large proportion of common SNPs with minor allele frequencies (MAF) > 10%. However, a much lower fraction of less common (5%–10% MAF) and rare (MAF < 5%) SNPs are captured by these arrays. The power of GWAS to detect variants with MAF < 10% is therefore limited. Additionally, GWAS arrays are not optimally formatted to capture indels and copy number variants, both of which are likely to have roles in disease susceptibility. New “exome chips” have recently been released that aim to address some of these limitations. However the success of these new arrays remains to be evaluated.

Since the completion of the human genome project in 2003, the utilisation of massively parallel sequencing technologies to identify variants has become feasible. Moreover, these methods can be used to detect small indels, substitutions and structural variants. Although in their infancy, such studies are beginning to identify additional variants. For example, highly-penetrant mutations have recently been identified in the proof reading domains of POLD1 (S478N) and POLE (L424V) in CRC families [36]. Another recent study identified 11 candidate CRC susceptibility genes with truncating mutations in two or more of 96 familial CRC cases [75]. To maximally utilise sequencing data, new bioinformatic techniques are required to remove sequencing errors and prioritise variants. Additionally, due to financial considerations such studies remain small and not powered to detect less common variants with moderate effects risk of disease. Using cases enriched for genetic susceptibility evidenced by a strong family history or early disease onset is a useful technique to increase the efficiency of these studies. In addition, utilising whole exome sequencing, as performed by Palles *et al.* [36], can dramatically reduce the costs associated with such projects. Coding variants are also much easier to interpret than those in non-coding regions. However, with every individual’s genome harboring 250–300 putative loss of function variants [76] and many missense variants being of unknown effect, identification of the disease causing variant presents a significant challenge. Recent studies are working to interpret this class of variants [77] and algorithms such as SIFT [78], PolyPhen2 [79] and CONDEL [80] aim to guide researchers by predicting the functional effects of coding mutations. Work to develop similar methods to deal with non-coding regions is still in its infancy [81,82], however, recent evidence [83,84] suggests that these regions are also *a priori* likely to contain variants with a large effect on CRC risk.

## 10. Conclusions

Much has been achieved in the study of genetic susceptibility to CRC in the last five decades. The architecture underlying this susceptibility is now recognised to be defined by a spectrum of predisposition alleles with different effect sizes and frequencies in the population. GWAS has proved a successful approach for identification of novel low-penetrance CRC risk alleles, improving our understanding of disease aetiology and providing novel therapeutic targets. Determining the biological processes underlying the associations at these loci presents a significant challenge and will likely require large collaborations between genetic researchers and functional biologists. Despite these advances, a large proportion of the heritability to CRC remains unaccounted for. It is hoped that the new generation of sequencing projects will help to uncover this missing heritability.
